# Radiomic Features Associated With HPV Status on Pretreatment Computed Tomography in Oropharyngeal Squamous Cell Carcinoma Inform Clinical Prognosis

**DOI:** 10.3389/fonc.2021.744250

**Published:** 2021-09-07

**Authors:** Bolin Song, Kailin Yang, Jonathan Garneau, Cheng Lu, Lin Li, Jonathan Lee, Sarah Stock, Nathaniel M. Braman, Can Fahrettin Koyuncu, Paula Toro, Pingfu Fu, Shlomo A. Koyfman, James S. Lewis, Anant Madabhushi

**Affiliations:** ^1^Center for Computational Imaging and Personalized Diagnostics, Case Western Reserve University, Cleveland, OH, United States; ^2^Department of Radiation Oncology, Taussig Cancer Center, Cleveland Clinic, Cleveland, OH, United States; ^3^Department of Otolaryngology and Head and Neck Surgery, University of Virginia, Charlottesville, VA, United States; ^4^Imaging Institute, Cleveland Clinic, Cleveland, OH, United States; ^5^Department of Population and Quantitative Health Sciences, Case Western Reserve University, Cleveland, OH, United States; ^6^Department of Pathology, Microbiology, and Immunology, Vanderbilt University Medical Center, Nashville, TN, United States; ^7^Louis Stokes Cleveland Veterans Administration Medical Center, Cleveland, OH, United States

**Keywords:** oropharyngeal squamous cell carcinoma, human papillomavirus, radiomics, prognosis prediction, nomograms

## Abstract

**Purpose:**

There is a lack of biomarkers for accurately prognosticating outcome in both human papillomavirus-related (HPV+) and tobacco- and alcohol-related (HPV−) oropharyngeal squamous cell carcinoma (OPSCC). The aims of this study were to i) develop and evaluate radiomic features within (intratumoral) and around tumor (peritumoral) on CT scans to predict HPV status; ii) investigate the prognostic value of the radiomic features for both HPV− and HPV+ patients, including within individual AJCC eighth edition-defined stage groups; and iii) develop and evaluate a clinicopathologic imaging nomogram involving radiomic, clinical, and pathologic factors for disease-free survival (DFS) prediction for HPV+ patients.

**Experimental Design:**

This retrospective study included 582 OPSCC patients, of which 462 were obtained from The Cancer Imaging Archive (TCIA) with available tumor segmentation and 120 were from Cleveland Clinic Foundation (CCF, denoted as S_CCF_) with HPV+ OPSCC. We subdivided the TCIA cohort into training (S_T_, 180 patients) and validation (S_V_, 282 patients) based on an approximately 3:5 ratio for HPV status prediction. The top 15 radiomic features that were associated with HPV status were selected by the minimum redundancy–maximum relevance (MRMR) using S_T_ and evaluated on S_V_. Using 3 of these 15 top HPV status-associated features, we created radiomic risk scores for both HPV+ (RRS_HPV+_) and HPV− patients (RRS_HPV−_) through a Cox regression model to predict DFS. RRS_HPV+_ was further externally validated on S_CCF_. Nomograms for the HPV+ population (M_p+RRS_) were constructed. Both RRS_HPV+_ and M_p+RRS_ were used to prognosticate DFS for the AJCC eighth edition-defined stage I, stage II, and stage III patients separately.

**Results:**

RRS_HPV+_ was prognostic for DFS for i) the whole HPV+ population [hazard ratio (HR) = 1.97, 95% confidence interval (CI): 1.35–2.88, *p* < 0.001], ii) the AJCC eighth stage I population (HR = 1.99, 95% CI: 1.04–3.83, *p* = 0.039), and iii) the AJCC eighth stage II population (HR = 3.61, 95% CI: 1.71–7.62, *p* < 0.001). HPV+ nomogram M_p+RRS_ (C-index, 0.59; 95% CI: 0.54–0.65) was also prognostic of DFS (HR = 1.86, 95% CI: 1.27–2.71, *p* = 0.001).

**Conclusion:**

CT-based radiomic signatures are associated with both HPV status and DFS in OPSCC patients. With additional validation, the radiomic signature and its corresponding nomogram could potentially be used for identifying HPV+ OPSCC patients who might be candidates for therapy deintensification.

## Introduction

The rise in the incidence of human papillomavirus (HPV)-related cancers has caused a significant epidemiological shift (1) in oropharyngeal squamous cell carcinoma (OPSCC). It is estimated that HPV causes more than 70% of OPSCC cases in the United States (2). HPV+ OPSCC differs from its HPV− counterpart in response to treatment and disease aggressiveness (3). In order to account for this, the most recent American Joint Committee on Cancer (AJCC) eighth edition tumor staging system was modified to incorporate HPV status, with different staging systems for HPV (p16) positive and negative tumors (4). HPV+ patients tend to respond better to definitive radiotherapy or combined chemoradiotherapy protocols and are less likely than HPV− patients to develop disease recurrence and metastases. Thus, it has become critical to develop biomarkers within the HPV+ and HPV− populations for risk stratification.

Treatment of OPSCC patients is on the cusp of a paradigm shift; current clinical trials are geared toward reducing treatment toxicity for HPV+ patients without compromising survival outcomes since low-risk patients typically could benefit from a lower dose of radiotherapy or less invasive surgical operations (treatment deintensification) (5). However, it is challenging to tailor the most optimal treatment strategy for each patient. Although high T (T3/T4) and N (N3) stages as well as tobacco use are clinically accepted risk factors for HPV+ OPSCC patients, these categorical predictors neglect the oncogenic differences between individual patients (6). A recent phase II randomized controlled trial NRG HN002 reported that patients who are grouped as candidates for treatment de-escalation using a combination of clinicopathologic factors did not meet the goal of 2-year disease-free survival (DFS) in the radiotherapy only arm (7). This exhibits a clear unmet clinical need for the development of objective biomarkers to identify patients who could truly benefit from treatment de-escalation.

On the other hand, the unmet need for HPV− patients is to precisely identify patients at high risk of developing local or regional failure after treatment, patients who might be candidates for targeted treatment escalation (8). While the eighth edition of the AJCC staging modifications that separate HPV+ and HPV− patients is a major advance (9), it still may not be sufficient for accurate risk stratification as it de-emphasizes the importance of nodal metastasis by diagnosing most of the new p16+ OPSCC patients into stages I and II (10).

Radiomics is the process of computational extraction of large numbers of quantitative imaging features, such as texture features, from routine radiologic scans (e.g., MRI and CT) for characterization of the disease (11). These features are able to detect subtle changes in imaging intensity patterns within a local region which in turn may help better describe the cancer phenotype as well as the tumor microenvironment. While radiomic features in the immediate vicinity outside the tumor have shown significant value in differentiating disease subtypes for lung (12) and breast (13) cancers, we are not aware of any work that has attempted to collectively evaluate the role of textural patterns from both within (intratumoral) and outside the tumor (peritumoral) to predict HPV status or to identify their association with disease-specific survival in OPSCC.

**Figure 1:**
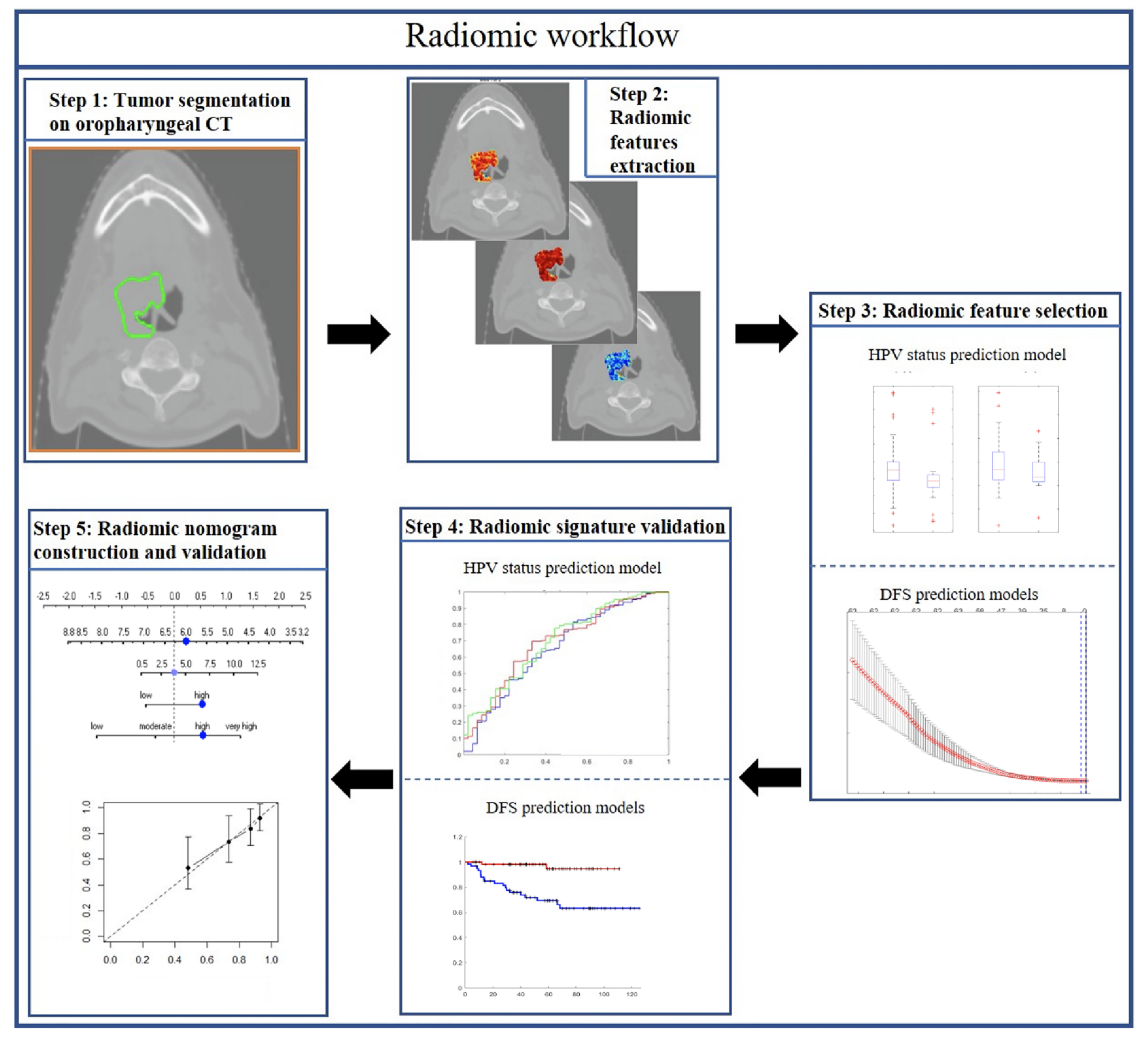
Diagram of the overall radiomic workflow.

In this study, we sought to explore the prognostic value of both intratumoral and peritumoral HPV status-associated radiomic features on CT scans and compared and combined them with clinical and pathologic factors on over 500 OPSCC patients. The prognostic radiomic biomarker for HPV+ OPSCC was validated both internally (237 patients) and externally (120 patients) on two different cohorts. We also evaluated the utility of the radiomic signature to prognosticate DFS within each individual AJCC eighth edition-defined stage group. Finally, this study also involved creation and validation of a clinicopathologic nomogram for estimating DFS for HPV+ OPSCC patients. **Figure 1** shows the overall methodology comprising radiomic feature extraction and selection, prognostic signatures, and radiomic nomogram construction and validation.

## Materials and Methods

### Patients

Two OPSCC cohorts were included in this study: The Cancer Imaging Archive (TCIA, *n* = 462) OPC-Radiomics cohort (10) and the Cleveland Clinic Foundation (CCF) cohort (*n* = 120). All patients had undergone pretreatment radiotherapy planning CT. TCIA is an open archive of deidentified cancer-specific medical images and associated clinical metadata accessible for public download (14). Clinicopathologic and outcome information for patients in the CCF cohort were collected after obtaining approval from the Institutional Review Board of Cleveland Clinic. Demographic data are shown in **Table 1**. For the TCIA cohort, a total of 473 patients with OPSCC treated with curative intent at the Princess Margaret Cancer Center between 2005 and 2010 were reviewed. Histopathologic confirmation was used for the diagnosis of OPSCC and p16 immunohistochemistry was used to assess HPV status. Patients were triaged using inclusion criteria that involved the availability of i) radiotherapy planning CT scans with matched clinical information (HPV status by p16 immunohistochemistry, survival information) and ii) binary mask for gross tumor volume (GTV). Fourteen patients with the following criteria were excluded: i) CT images containing artifact (*n* = 6); ii) number of voxels within tumor is less than 200, which was deemed to be insufficient for feature extraction (*n* = 5); and iii) tumor mask contains normal brain tissue (*n* = 3). Following the patient exclusion criteria, 462 patients from the TCIA cohort and 120 patients from the CCF cohort were included for subsequent radiomic analysis. The flowchart for patient enrollment is illustrated in **Supplementary Figure S1**.

**Table 1:** Clinicopathologic data for HPV+ and HPV− patients included in this study.

Clinical parameter	Patient demographics						
	HPV+ patients				HPV− patients		
	TCIA training (S_T_, *n* = 100)	TCIA validation (S_V_, *n* = 237)	CCF validation (S_CCF_, *n* = 120)	*p-*value	TCIA training (S_T_, *n* = 80)	TCIA validation (S_V_, *n* = 45)	*p-*value
Age	60.4 ± 9.07	58.3 ± 9.43	59.45 ± 9.52	0.11	65.54 ± 10.39	64.87 ± 9.63	0.63
Gender							
Male	82 (82%)	197 (83.1%)	108 (90%)	0.16	58 (72.5%)	31 (68.9%)	0.82
Female	18 (18%)	40 (16.9%)	12 (10%)		22 (27.5%)	14 (31.1%)	
Smoking history							
Non-smoker	41 (41%)	83 (35%)	47 (35%)		5 (6.3%)	7 (15.6%)	0.18
Ex-smoker	39 (39%)	95 (40.1%)	51 (40.1%)	0.59	30 (37.5%)	18 (40%)	
Current	20 (20%)	59 (24.9%)	22 (24.9%)		45 (56.2%)	20 (44.4%)	
Drinking history							
Non-/light drinker	82 (82%)	191 (80.6%)	102 (85%)	0.39	39 (48.8%)	22 (48.9%)	0.70
Ex-drinker	6 (6%)	12 (5.1%)	9 (7.5%)		9 (11.2%)	9 (20%)	
Heavy drinker	12 (12%)	34 (14.3%)	9 (7.5%)		32 (40%)	14 (31.1%)	
T stage							
T1	23 (23%)	45 (19%)	27 (22.5%)	0.35	7 (8.7%)	3 (6.7%)	0.91
T2	36 (36%)	81 (34.2%)	46 (38.3%)		23 (28.8%)	15 (33.3%)	
T3	30 (30%)	65 (27.4%)	24 (20%)		30 (37.5%)	15 (33.3%)	
T4	11 (11%)	46 (19.4%)	23 (19.2%)		20 (25%)	12 (26.7%)	
N stage							
N0	13 (13%)	26 (11%)	12 (10%)	0.93	23 (28.7%)	14 (31.1%)	0.99
N1	53 (53%)	132 (55.7%)	73 (60.8%)		32 (40%)	18 (40%)	
N2	27 (27%)		29 (24.2%)		19 (23.8%)	10 (22.2%)	
N3	7 (7%)	57 (24.1%)22 (9.2%)	6 (5%)		6 (7.5%)	3 (6.7%)	
Overall stage (AJCC eighth edition)							
I	42 (42%)	98 (41.4%)	62 (51.7%)	0.07	22 (27.5%)	13 (28.9%)	0.97
II	41 (41%)	76 (32%)	30 (25%)		32 (40%)	17 (37.8%)	
III	17 (17%)	63 (26.6%)	27 (22.5%)		26 (32.5%)	15 (33.3%)	
IV	0 (0%)	0 (0%)	1 (0.8%)		0 (0%)	0 (0%)	

### CT Imaging

The CT images for the TCIA cohort were acquired (10) from one of the following CT scanners: General Electric Discovery ST, General Electric Lightspeed Plus, or Toshiba Medical Systems Aquillion ONE. CT scans were acquired in helical mode with a slice thickness of 2.5 mm (General Electric) or 2 mm (Toshiba), at 120 kVp and 300 mAs tube current. Image resolution was 1 mm for all the scans. The CT images for the CCF cohort were acquired from either The General Electric Medical System or The Siemens Medical System. CT scans were acquired in helical mode with a slice thickness of 3 mm, at 120 kVp and 235 mAs tube current. Image resolution is between 0.4 and 0.5 mm for most of the patients, with an image matrix of 512 × 512.

### Intratumoral and Peritumoral Compartment Definitions

The binary intratumoral masks which outlined the primary GTV were obtained using the Radiation Therapy Structures (RTSTRUCT) for the TCIA cohort (10). Primary tumors on the CCF cohort were manually segmented by two board-certified head and neck radiologists JL (with 5 years of clinical expertise) and SS (with 6 years of clinical expertise) across all of the two-dimensional CT sections using a hand-annotation tool in axial view. Morphologic dilation operations were then performed for all patients on intratumoral masks to define the annular ring region outside the tumor up to a radial distance of 15 mm based on previous studies in lung (15) and breast cancer (13), where peritumoral margins >15 mm were not associated with disease recurrence. The intratumoral masks were then subtracted from the dilated masks to obtain the peritumoral regions, which were then subdivided into three peritumoral rings of 5-mm-radius increments. Implementation details on peritumoral masks are provided in **Appendix E1**, section 1.

### Radiomic Feature Extraction

A total of 664 intratumoral and 1,485 peritumoral (495 × 3 peritumoral rings) radiomic features were extracted for all patients on all the compartments on a per-pixel basis. The feature sets for each study utilized included 16 gray-level intensity features (quantifying statistics of the raw intensity within a specific window size of 3 × 3, 5 × 5, 7 × 7, and 9 × 9), 40 intensity gradient-based features (quantifying intensity gradient variability), 52 gray-level co-occurrence matrix (GLCM) Haralick features (capturing disorder patterns of the adjacent pixel intensities within local pixel neighborhoods) (16), 20 Laws energy (capturing combinations of five irregular texture enhancement patterns: levels, spots, edges, waves and ripples in an image) (17), 28 Gabor wavelet-based features (capturing structural detail at seven orientations of 22.5°, 45°, 67.5°, 90°, 112.5°, 135°, 157.5°, and 4 scales of 2, 4, 8, and 12 pixels) (18), and 52 CoLlAGe features (capturing textural heterogeneity by applying GLCM metrics to local anisotropic gradient orientations) (19). All of these texture features were extracted in both intratumoral and peritumoral compartments (0–5, 5–10, and 10–15 mm) on all slices containing the tumor. Statistics of mean, median, standard deviation, skewness, and kurtosis were calculated from the feature responses of all pixels within the region of interest. A list of the extracted features is summarized in **Supplementary Table S1**, with their detailed descriptions provided in **Appendix E1**, section 2. All feature values were transformed into new scores with a mean of 0 and a SD of 1 (*z*-score transformation). Both intratumoral and peritumoral feature extraction pipelines are now publicly available at https://github.com/ccipd.

### Statistical Analysis

Within the TCIA cohort, 462 patients were randomly allocated to S_T_ (180 patients: 100 HPV+ and 80 HPV−) and S_V_ (282 patients: 237 HPV+ and 45 HPV−) in an approximately 3:5 ratio. For both HPV+ and HPV− patients, the ratio of non-censored patients in S_T_ and S_V_ was kept balanced. The clinical end points of interest for this study were HPV status and DFS. DFS was defined as the time interval from the radiotherapy end date to the date of either last follow-up (censored) or local, regional, distant failure and death (event), whichever happened first. The difference of continuous variables (i.e., age) between cohorts (i.e., S_T_, S_V_, and S_CCF_) was determined using the analysis of variance (ANOVA) and the association between categorical factors was estimated using the chi-square test.

Since there were no HPV− patients in the CCF cohort, we used only the TCIA cohort for HPV status prediction. A machine learning classifier was first constructed using a combination of intratumoral and peritumoral features for the prediction of HPV status using S_T_. To remove redundant features, all possible pairs of features in S_T_ were tested for correlation by calculating the Spearman correlation coefficient (SCC). For any pair of features with SCC greater than 0.80, the feature with the higher Wilcoxon rank sum *p*-value was removed. A linear discriminant analysis (LDA) machine-learning classifier was subsequently trained in conjunction with the minimum redundancy–maximum relevance (MRMR) (20) feature selection approach using a 100-run, 3-fold cross-validation setting. The top 15 most frequently selected radiomic features (F_t_) that best discriminated between HPV+ vs. HPV− across all iterations were identified from S_T_. An unadjusted *p*-value <0.05 (using two-sided Wilcoxon rank sum tests) was employed to indicate statistical significance. These features were then evaluated *via* the LDA classifier in terms of HPV status prediction on S_V_ using the area under the receiver operating characteristic curve (AUC) metric.

We also developed dedicated radiomic risk score classifiers for patients within the individual HPV+ (RRS_HPV+_) and HPV− (RRS_HPV−_) categories. The least absolute shrinkage and selection operator (LASSO) method was applied to F_t_ using the cases in S_T_ for both HPV+ and HPV− patients. After identifying the top ranked features, the corresponding LASSO coefficients were used for constructing risk classifiers for the HPV+ (RRS_HPV+_) and HPV− (RRS_HPV−_), respectively. Both RRS_HPV+_ and RRS_HPV−_ were calculated for each patient *via* a linear combination of selected features that were weighted by corresponding coefficients:

$$\text{RR}{\text{S}}_{\text{HPV}+/-}=\Sigma_{i=1}^{n}\;\beta_{i}*x_{i}$$

where *n* (ranging from 0 to 15) is the number of features selected by LASSO for HPV+ or HPV− patients, *x_i_* refers to the HPV status-associated feature value, and *β_i_* is the corresponding weighted coefficient. The potential association of RRS_HPV+_ and RRS_HPV−_ with DFS was first assessed in S_T_ and then evaluated in S_V_. The prognostic ability of RRS_HPV+_ was further externally validated in S_CCF_. Patients were classified into high or low risk based on the median value of the RRS_HPV+_ or RRS_HPV−_ in S_T_, which was then applied to S_V_ and S_CCF_. Kaplan–Meier survival curves were used to visualize the survival rate for the high- and low-risk groups. At any given point on the survival curve, the probability that a patient in either the high-risk or low-risk group remains alive is presented (21). The log-rank test and hazard ratio were used to compare the survival differences between the two groups. The same Kaplan–Meier survival analyses were further performed for each cancer stage group defined by the AJCC eighth edition on the dataset combining S_V_ and S_CCF_. The final values for *n* were determined when the hazard ratio for high risk over low risk reached the highest in S_V_ for HPV+ patients (**Supplementary Figure S4**). The value of the tuning parameter in the LASSO-Cox model (*λ*) was averaged out by 10 cross-validation to minimize the error within S_T_. Constructions of RRS_HPV+_ and RRS_HPV−_ were performed using in-house software implemented in the MATLAB R2019b platform (MathWorks).

Univariate Cox proportional hazards analysis on the effect of RRS_HPV+_, RRS_HPV−_, and the individual clinicopathologic variables (gender, smoking status, drinking status, T stage, N stage, and AJCC eighth edition of overall stage) on DFS was evaluated. Variables significant in univariate analysis were included for multivariable Cox proportional hazards analysis to investigate the relationships between the various covariates (including the RRS_HPV+_, RRS_HPV−_, and the clinicopathologic variables).

To further investigate the independent prognostic value of the RRS_HPV+_ with existing clinical factors (gender, smoking, and drinking status) and pathological staging factors (T stage, N stage, and the AJCC stage eighth edition), we constructed nomogram models for HPV+ patients comprising i) only the clinical factors (M_c_) of gender, smoking, and drinking history; ii) only the pathologic staging factors (M_p_) of AJCC eighth edition overall stage and N stage; and iii) both the pathologic T stage and the RRS_HPV+_ (M_p+RRS_) using S_T_ and validated them in S_V_ and S_CCF_. The prognostic ability of M_p+RRS_ was compared against M_c_ and M_p_ for HPV+ populations in terms of the concordance index (C-index). The nomograms were validated using 1,000 bootstrap resampling to calculate C-index with confidence intervals. Calibration curve analysis was performed to compare the nomogram-predicted DFS with the actual DFS. Decision curve analysis was adopted to calculate the net benefit for M_p+RRS_ in comparison with M_c_ and M_p_, for verification of the clinical usefulness of the nomogram (22). Nomogram construction, calibration plot, and decision curve were implemented using the “rms” and “SvyNom” packages under R statistical software (version 4.0.3; R Foundation for Statistical Computing, Vienna, Austria).

## Results

### Clinicopathologic Characteristics

The clinical and pathologic characteristics of patients in the TCIA training set (S_T_), TCIA internal validation set (S_V_), and CCF external validation set (S_CCF_) are summarized in **Table 1**. No significant differences were found in most features among S_T_, S_V_, and S_CCF_. All patients included in this study underwent radiotherapy. Eighty-seven of the 180 patients in S_T_ (48.3%) and 145 of the 282 patients in S_V_ (51.4%) were also treated with chemotherapy. The median DFS for HPV+ patients was 6.19 years in S_T_ and 6.24 years in S_V_. The median DFS for HPV− patients was 1.27 years in S_T_ and 1.55 years in S_V_. Thirty-three percent (33/100, 6 local) and 33% (79/237) of HPV+ patients were not censored in S_T_ and S_V_, respectively, while 77.5% (62/80) and 68.9% (31/45) of HPV− patients were not censored in S_T_ and S_V_, respectively. There were 62 (18.4%) recurrences (15 local, 8 regional, and 39 distant) for HPV+ patients and 64 (51.2%) recurrences (30 local, 11 regional, and 23 distant) for HPV− patients.

### Experiment 1: Prediction of HPV Status

Within S_T_, 454 uncorrelated intratumoral and peritumoral features were obtained after feature pruning. From these uncorrelated features, the top 15 were identified for predicting HPV status by MRMR feature selection, of which 11 were peritumoral features and 4 were intratumoral features (**Table 2**).

**Table 2:** Top 15 features for HPV status prediction and notation of involvement in HPV status-specific prognostic prediction in experiment 2.

Feature names (parameters)	ROI	Rank sum *p-*value (unadjusted)	Contribute to DFS prediction for HPV+?	Contribute to DFS prediction for HPV−?
CoLlAGe (Std of sum-variance)	Peritumoral 0–5 mm	3.0 × 10^−7^	✘	✘
Haralick (mean of Info1)	Peritumoral 0–5 mm	1.3 × 10^−8^	✘	✘
Gabor (median, *λ* = 3, *θ* = 0.1 rad)	Peritumoral 5–10 mm	8.5 × 10^−6^	✓	✓
CoLlAGe (skewness of sum-average)	Peritumoral 5–10 mm	0.0046	✘	✓
CoLlAGe (kurtosis of diff-average)	Peritumoral 0–5 mm	1.78 × 10^−4^	✘	✘
Haralick (Std of Info1)	Peritumoral 5–10 mm	4.7 × 10^−4^	✘	✘
Gabor (skewness, *λ* = 5, *θ* = 0.0 rad)	Peritumoral 10–15 mm	0.001	✘	✘
Laws (median of E5L5)	Peritumoral 10–15 mm	0.0335	✘	✘
Laws (skewness L5R5)	Peritumoral 0–5 mm	6.5 × 10^−4^	✘	✘
Gabor (kurtosis, *λ* = 3, *θ* = 0.6 rad)	Peritumoral 0–5 mm	4.8 × 10^−6^	✘	✓
CoLlAGe (Std of sum-average)	Peritumoral 0–5 mm	5.3 × 10^−5^	✓	✘
Haralick (median of info1)	Intratumoral	1.8 × 10^−5^	✘	✘
CoLlAGe (skewness of diff-variance)	Intratumoral	4.4 × 10^−4^	✘	✘
Gray (median of mean intensity)	Intratumoral	0.001	✓	✘
Haralick (skewness of diff-variance)	Intratumoral	0.005	✘	✘

**Figure 2:**
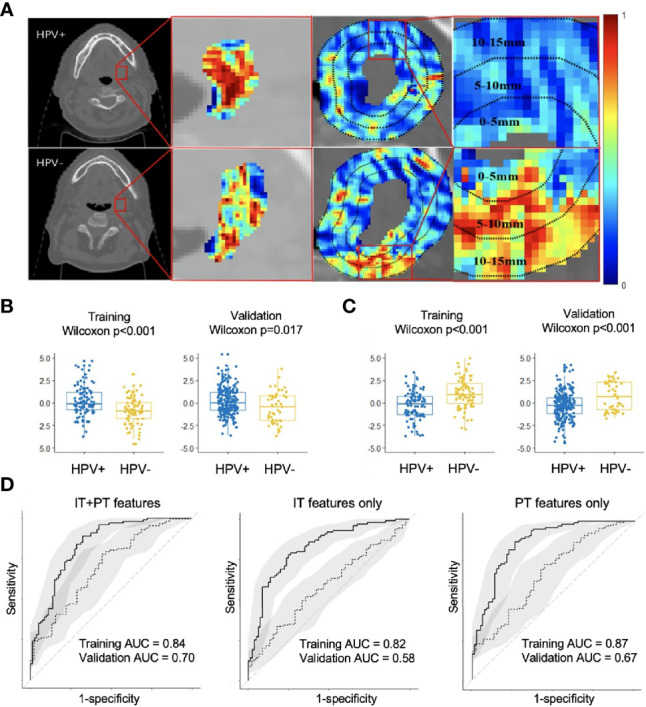
Feature map for the best intratumoral and peritumoral features expressing differently on the example HPV+ and HPV− CT slices overlaid with either tumor or annular ring areas around the tumor **(A)**. Boxplots showing distribution differences for the best intratumoral **(B)** and peritumoral feature **(C)** between HPV+ and HPV− patients in both training (S_T_) and validation (S_V_). Receiver operating characteristic (ROC) analysis of radiomic features for predicting HPV status on training (S_T_, *n* = 180) and validation (S_V_, *n* = 282) cohorts with confidence intervals **(D)**. Using combined intratumoral and peritumoral features yielded the best result in S_V_. IT, intratumoral; PT, peritumoral.

Changes in classification performance on account of differently selected features in S_T_ are provided in **Supplementary Figure S2**. Standard deviation of CoLlAGe sum of variance from the 0–5-mm rings (**Figure 2C**) was identified as the most discriminating peritumoral feature (*p* < 0.001), while the median of Haralick correlation-info1 (**Figure 2B**) was identified as the most discriminating feature (*p* < 0.001) within the tumor. Both features were differentially expressed on CT scans between HPV+ and HPV− patients, and representative examples are illustrated *via* colormaps of the feature representations overlaid on the tumor areas or the annular ring regions around the tumor (**Figure 2A**). Noticeably, three of the four selected Haralick features were measures of correlation applied with different statistics on both intratumoral and peritumoral regions, suggesting that there might be significant pixel correlation-related pattern differences between the HPV+ and HPV− patients. We also observed consistently higher Laws feature values for HPV− patients compared with HPV+ across S_T_ and S_V_. These features quantify intensity smoothness, abrupt edge changes, and ripple patterns on CT scans. Interestingly, all the selected Laws and Gabor features were from regions outside the tumor. Further details on the selected features are provided in **Supplementary Figure S3**.

The areas under the curve (AUCs) for using intratumoral and peritumoral alone on S_T_ are 0.82 (95% CI: 0.76, 0.88) and 0.87 (95% CI: 0.81, 0.92), respectively. The corresponding AUCs on S_V_ are 0.58 (95% CI: 0.49, 0.67) and 0.67 (95% CI: 0.58, 0.76). When combining the intratumoral and peritumoral features, ROC analysis on S_T_ yielded an accuracy of 0.79 and AUC of 0.84 (95% CI: 0.78, 0.90), with a sensitivity of 0.89 and specificity of 0.68 when using a threshold of 0.4. For S_V_, we obtained an accuracy of 0.74 and an AUC of 0.70 (95% CI: 0.62, 0.79), with a sensitivity of 0.78 and specificity of 0.53 when the same threshold from S_T_ was applied. **Figure 2D** illustrates that using combined peritumoral and intratumoral features improved AUC compared with only using the intratumoral features and using only the peritumoral features for predicting HPV status.

### Experiment 2: Prognosticate DFS for Both HPV− and HPV+ Patients, Including Within Individual AJCC Eighth Edition-Defined Stage Groups

**Figure 3:**
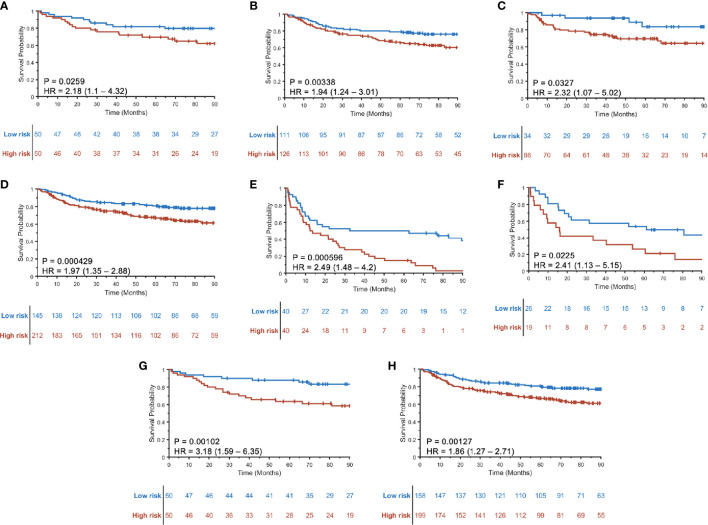
Kaplan–Meier curves for disease-free survival (DFS) using RRS_HPV+_ in training S_T_ **(A)**, internal validation S_V_ **(B)**, external validation S_CCF_ **(C)**, and the combined validation set S_V_+S_CCF_ **(D)**. Kaplan–Meier curves for DFS prediction using RRS_HPV−_ in S_T_ **(E)** and S_V_ **(F)**. DFS prediction for HPV+ patients in the S_T_ **(G)** and S_V_+S_CCF_ set **(H)** using radiomic nomogram (M_p+RRS_), which contains pathologic tumor stage and the RRS_HPV+_.

A three-feature radiomic signature was identified as having the best prediction of DFS for both HPV+ and HPV− patients (**Supplementary Figure S4**). The radiomic risk scores for HPV+ patients (RRS_HPV+_) and HPV− patients (RRS_HPV−_) across S_T_, S_V_, and S_CCF_ are illustrated in **Supplementary Figure S5**. Details of the features selected are provided in **Table 2**, with their coefficients in the Cox model provided in **Supplementary Figure S6**. RRS_HPV+_ was constructed using two peritumoral and one intratumoral features, while RRS_HPV−_ was constructed using three peritumoral features. The median RRS_HPV+_ value (−0.0809) in S_T_ was used as the cutoff threshold for defining high- and low-risk groups, resulting in statistically significant DFS prediction by KM analysis in S_T_ (log-rank test, *p* = 0.026, HR = 2.18), S_V_ (log-rank test, *p* = 0.003, HR = 1.94), S_CCF_ (log-rank test, *p* = 0.033, HR = 2.32), and S_V_+S_CCF_ (log-rank test, *p* < 0.001, HR = 1.97) as illustrated in **Figures 3A–D**, respectively. The median RRS_HPV−_ value (0.0076) in S_T_ also resulted in a statistically significant prediction of DFS by Kaplan–Meier analysis in both the S_T_ (log-rank test, *p* < 0.001, HR = 2.49) and S_V_ (log-rank test, *p* = 0.023, HR = 2.41), as illustrated in **Figures 3E, F**, respectively.

**Figure 4:**
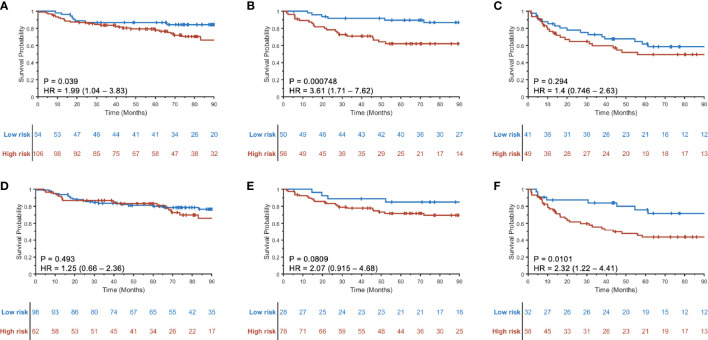
Kaplan–Meier curves for prognostication using RRSHPV+ within the AJCC eighth edition-defined overall stage I **(A)**, II **(B)**, and III **(C)** HPV+ OPSCC patients. Similarly, Kaplan–Meier curves using the radiomic nomogram M_p+RRS_ for prognostication within overall stage I **(D)**, II **(E)**, and III **(F)** HPV+ OPSCC patients.

The prognostic ability of the radiomic risk score (RRS_HPV+_) was further evaluated for the patients within the AJCC eighth edition-defined different stage groups in S_V_+S_CCF_. The HRs of predicting DFS using RRS_HPV+_ for stage I (**Figure 4A**), stage II (**Figure 4B**), and stage III (**Figure 4C**) HPV+ patients were 1.99 (95% CI: 1.04–3.83, *p* = 0.039), 3.61 (1.71–7.62, *p* < 0.001), and 1.4 (0.746–2.63, *p* = 0.294), respectively.

Results of the univariable analysis are shown in **Table 3**. T3 stage, a moderate or heavy drinking history, and a higher RRS_HPV+_ were significantly associated with worse DFS for the HPV+ population in S_T_. N2 stage and a higher RRS_HPV−_ were significantly associated with worse DFS for the HPV− population in S_T_. In multivariable analysis, RRS_HPV+_ (DFS hazard ratio, 30.12, 95% CI: 5.67–159.96, *p* < 0.001) and T3 stage (DFS hazard ratio, 2.94, 95% CI: 1.02–8.45, *p* = 0.04) remained independent prognostic factors for HPV+ patients in the Cox proportional hazards model (**Table 4**). For HPV− patients, RRS_HPV−_ (DFS hazard ratio, 3.37, 95% CI: 1.93–5.88, *p* < 0.001), N1 stage (DFS hazard ratio, 2.08, 95% CI: 1.06–4.07, *p* = 0.03), and N2 stage (DFS hazard ratio, 2.55, 95% CI: 1.21–5.36, *p* = 0.01) were the independent prognostic factors in the multivariable Cox proportional hazards model.

**Table 3:** Univariable Cox proportional hazard model analysis in the training set (S_T_) for HPV+ and HPV− patients.

Variables	HPV+ patients		HPV− patients	
	HR (95% CI)	*p-*value	HR (95% CI)	*p-*value
Gender (female *vs.* male)	1.32 (0.78–1.84)	0.41	1.12 (0.63–1.86)	0.97
Smoking history				
(current smoker *vs.* non-/ex-smoker)	1.36 (0.54–3.39)	0.39	1.04 (0.63–1.73)	0.87
Drinking history				
(moderate/heavy drinker *vs.* non-/ex-/light drinker)	1.66 (1.05–2.42)	**0.02**	1.21 (0.72–2.05)	0.47
T stage				
T1	Ref		Ref	
T2	1.26 (0.42–3.78)	0.82	0.88 (0.32–2.39)	0.80
T3	2.9 (1.03–8.15)	**0.03**	1.11 (0.42–2.91)	0.83
T4	2.9 (0.84–10.13)	0.09	1.17 (0.42–3.22)	0.76
N stage				
N0	Ref		Ref	
N1	0.71 (0.26–1.96)	0.51	2.12 (0.89–4.13)	0.14
N2	0.82 (0.27–2.46)	0.72	2.61 (1.25–5.41)	**0.01**
N3	1.47 (0.35–6.20)	0.59	2.40 (0.85–6.75)	0.10
Overall stage (AJCC eighth edition)				
I	Ref		Ref	
II	1.81 (0.82–3.99)	0.14	1.02 (0.55–1.90)	0.94
III	2.19 (0.85–5.68)	0.11	1.27 (0.66–2.42)	0.47
RRS_HPV+_				
(RRS_HPV−_)	29.45 (7.2–120)	**<0.0001**	6.28 (2.06–19.16)	**0.0007**

Bold values refer to significant p values < 0.05.

**Table 4:** Multivariable Cox proportional hazard model analysis in the training set (S_T_) for HPV+ and HPV− patients.

HPV+ patients			HPV− patients		
Variables	HR (95% CI)	*p-*value	Variables	HR (95% CI)	*p-*value
Drinking history (moderate/heavy drinker *vs.* non-/ex-/light drinker)	0.81 (0.36–1.82)	0.61	N stage		
			N0	Ref	
			N1	2.08 (1.06–4.07)	**0.03**
			N2	2.55 (1.21–5.36)	**0.01**
			N3	2.80 (0.98–7.97)	0.054
T stage			RRS_HPV−_	3.37 (1.93–5.88)	**<0.0001**
T1	Ref				
T2	1.22 (0.38–3.92)	0.74			
T3	2.94 (1.02–8.45)	**0.04**			
T4	2.56 (0.70–9.35)	0.16			
RRS_HPV+_	30.12 (5.67–159.96)	**<0.0001**			

Bold values refer to significant p values < 0.05.

### Experiment 3: Using Radiomic Nomogram M_p+RRS_ to Prognosticate DFS Among HPV+ OPSCC

**Figure 5:**
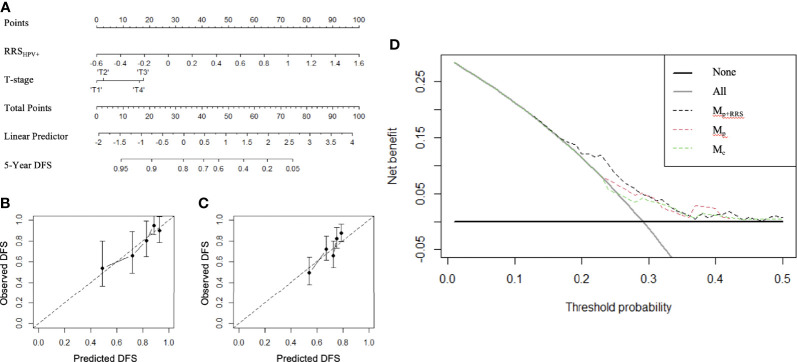
The constructed radiomic nomogram M_p+RRS_ **(A)** integrating the pathologic tumor stage (T stage) with the RRS_HPV+_. RRS_HPV+_ has more effect on DFS than the T stage, as indicated by a wider range of the total points. Calibration curves have good agreement between predicted and actual survival probability on S_T_ **(B)** and S_V_+S_CCF_ **(C)**. Decision curve on S_T_+S_V_+S_CCF_ **(D)** compared the clinical usefulness of radiomic nomogram M_p+RRS_ (black dash line) in DFS prediction against the pathologic staging nomogram M_p_ (red dash line) and the clinical nomogram M_c_ (green dash line).

Variables significant in both univariable and multivariable analyses (pathologic T stage and RRS_HPV+_) were used to develop the radiomic nomogram M_p+RRS_ (**Figure 5A**) for HPV+ patients. The calibration curve of M_p+RRS_ for estimating DFS showed good agreement between the predicted and the observed survival probability in both S_T_ (**Figure 5B**) and the combined validation set S_V_+S_CCF_ (**Figure 5C**). The C-index of M_p+RRS_ for estimating DFS in S_T_ was 0.72 (95% CI: 0.62–0.81), while the C-index for the pathologic staging nomogram M_p_ was 0.62 (95% CI: 0.51–0.72), and that for the nomogram M_c_ using gender, smoking, and drinking history was 0.59 (95% CI: 0.49–0.69). When evaluated on S_V_+S_CCF_, M_p+RRS_ yielded a C-index of 0.59 (95% CI: 0.54–0.65) for DFS prediction, while for M_p_ and M_c_, the C-indices were 0.59 (95% CI: 0.53–0.64) and 0.56 (95% CI: 0.51–0.61), respectively. The Kaplan–Meier survival curves for M_p+RRS_ in S_T_ and in S_V_+S_CCF_ are shown in **Figures 3G, H**. In addition, M_p+RRS_ was significantly associated with DFS, independent of M_c_ and M_p_ in the multivariable analysis when evaluated on S_T_+S_V_+S_CCF_ (**Table 5**). In the decision curve analysis, M_p+RRS_ yielded a better net benefit compared with M_c_ or M_p_ individually when the threshold probability <0.35 (**Figure 5D**).

**Table 5:** Comparison between the radiomic nomogram M_p+RRS_, the pathologic staging nomogram M_p_, and the clinical factors nomogram M_c_ for DFS prediction in S_T_+S_V_+S_CCF_.

M_p+RRS_, M_c_, and M_p_ (S_T_+S_V_+S_CCF_, *N* = 457, 136 died or had recurrence)				
Model	HR (95% CI)	C-index (95% CI)	Univariable *p*-value	Multivariable *p*-value
M_p+RRS_	1.6 (1.4–2)	0.62 (0.57–0.67)	<0.001***	<0.001***
M_p_	2.2 (1.4–3.5)	0.59 (0.55–0.64)	<0.001***	0.057
M_c_	2.1 (1.4–3)	0.57 (0.52–0.61)	<0.001***	0.072

***refers to extreme significant p values < 0.001.

When evaluating the prognostic ability of M_p+RRS_ for HPV+ patients in S_V_+S_CCF_ within each AJCC eighth edition-defined stage group, univariable Cox proportional hazard regression yielded HRs of 1.25 (95% CI: 0.66–2.36, *p* = 0.493) for stage I, 2.07 (0.915–4.68, *p* = 0.081) for stage II, and 2.32 (1.22–4.41, *p* = 0.01) for stage III patients. The corresponding KM curves are shown in **Figures 4D–F**.

## Discussion

HPV+ OPSCC has better clinical prognosis and treatment response than the alcohol- and tobacco-related HPV− OPSCC (23). Because of this, treatment deintensification to reduce therapy-related morbidity in “low-risk” HPV+ OPSCC patients is being considered. However, a recent phase II randomized controlled trial by Yom et al. reported that patients in whom therapy was deintensified based on traditional TNM staging information did not meet the goal of 2-year DFS >85% (7). This was likely due to the lack of biomarkers for identifying patients who would most benefit from therapy deintensification. Although the AJCC eighth edition staging system represents a major improvement over the seventh edition, better and more reliable methods for pretreatment prognostication are needed for therapeutic decision-making. The radiomic biomarkers presented in this study aimed to identify those truly low-risk HPV+ patients within both the whole and AJCC eighth edition stage 1 population groups, for whom treatment deintensification should be considered.

In this work, we investigated the ability of both intratumoral and peritumoral radiomic biomarkers on CT scans to predict HPV status for a large cohort of 462 OPSCC patients. Additionally, we addressed the OPSCC prognosis prediction problem independently within the HPV+ and HPV− populations. The radiomic signature which was prognostic for the whole HPV+ population was also prognostic within the AJCC eighth edition stage I and stage II patients. Finally, the radiomic features were combined with pathologic staging factors to form a radiomic nomogram for individualized prognosis estimation for HPV+ OPSCC patients.

Currently, measuring p16 protein expression *via* immunohistochemistry is the recommended test for determining HPV status. However, distinct populations of patients exist in whom the tumors overexpress p16 but are in fact negative for HPV-DNA or mRNA expression and vice versa (24). Consequently, using p16 testing alone to determine HPV status results in some misclassified patients. Additional biomarkers are needed to complement the p16 testing. In the first experiment, we demonstrated that radiomic features from within the tumor and annular rings of 0–15 mm outside the tumor on CT imaging could reasonably predict HPV status of OPSCC, with an overall accuracy of 76%. A recent study by Leijenaar et al. designed a statistical framework for HPV status prediction, and they found that HPV+ tumors are more homogeneous in CT densities (25). This finding is in alignment with the result of this study. Specifically, HPV+ tumors possess a more homogeneous morphologic appearance in terms of CT texture patterns compared with HPV− tumors, which in turn is characterized by the Haralick correlation of information measured within the tumor (**Figure 2A**, second column). A higher value of the Haralick correlation indicates less pixel intensity disorders and decreased morphologic appearance heterogeneity for HPV+ tumors compared with HPV− tumors. Similar to the findings we report, Bagher-Ebadian et al. reported that HPV+ tumors have consistently lower energy components for seven frequency bands quantified by the DOST features (26). Although the discriminating textural features we identified are different from previous studies, the interpretations of the features are similar. However, we also found higher gray-level intensity values of HPV+ tumors compared with HPV− tumors on CT, which appears to be at odds to the findings of the study by Leijenaar et al. (25). In addition, our study represents the first study for integrating CT radiomic both within and around the tumor for OPSCC HPV status prediction. We demonstrate the superior discriminability of peritumoral CT radiomic features, which appears to suggest discriminable differences of the microenvironment in the regions immediately outside the tumor. HPV− patients are best characterized by a combination of local intensity disorder and microscale heterogeneity in gradient orientation, particularly outside the tumor. Specifically, a higher peritumoral variation of the gradient orientation defined by the CoLlAGe sum of variance was observed in HPV− compared with HPV+ tumors. Reduced expression of Haralick information capturing low level of correlation between adjacent pixels was also an important component of the peritumoral radiomic signature for HPV− patients. Furthermore, HPV− peritumoral regions were characterized by abrupt changes in edges, ripples, and intensity smoothness, as detected by the elevated expression of Laws features. These findings are consistent with previous studies, which showed that HPV+ tumors have less stroma overall, have smoother borders to the nests and leading edges, and are more homogeneously cellular usually without keratin production (27–29).

In the second experiment, we evaluated the prognostic ability of the HPV status-associated radiomics features found in experiment 1, to separately stratify HPV+ and HPV− OPSCC patients into high- and low-risk groups based on DFS. We constructed dedicated radiomic risk scores (RRS_HPV+_, RRS_HPV−_), which yielded significant risk stratification based on DFS in the validation set for both HPV+ and HPV− populations. A previous study by Leijenaar et al. externally validated the prognostic value of intratumoral radiomic signatures in a larger cohort of 542 OPSCC (C-index = 0.628, *p* < 0.001) but did not consider the variation of the results by HPV status (30). Vallières et al. used radiomic features from pretreatment FDG-PET and CT images of 300 patients from four different cohorts to prognosticate outcomes in head and neck cancer (31). They obtained C-indices of 0.63, 0.88, and 0.60 for local, regional, and distant recurrence-free survival. Aerts et al. trained a prognostic radiomic model on 422 patients with lung cancer and validated on 231 patients with head and neck cancers (32), achieving a concordance index of 0.69 (*p* < 0.001) on the validation set. However, unlike our study, this study did not consider HPV status as an independent prognostic indicator for outcome prediction. The prognostic ability of the radiomic risk score (RRS_HPV+_) was also evaluated within each of the AJCC eighth edition-defined stage groups. Although various treatment deintensification strategies have been proposed by multiple clinical trials based on clinicopathologic factors, there is a lack of reliable biomarkers for risk-stratifying OPSCC patients within individual stage groups. In the HN002 trial, patients meeting criteria of either T1-T2 N1-N2b M0 or T3 N0 N-2b M0 (AJCC seventh ed.) with a ≤10 pack-year smoking history are selected as candidates for therapy deintensification (7). Their results showed that patients randomly assigned to the non-chemotherapy arm did not meet the goal of 2-year DFS >85%. The authors also reported that for these patients using a low-than-standard-dose radiotherapy resulted in a higher rate of locoregional failure. This indicates that risk stratification based on grouping of clinicopathologic factors alone is not robust and there is a clear unmet clinical need for developing more granular and more objective biomarkers to identify patients who could truly benefit from treatment de-escalation. Clinically, stage I and stage II patients are the current target for treatment de-escalation. However, a subset of these patients still had poor survival outcome and would not benefit from treatment de-escalation (33). The CT radiomic risk score developed in this study represents a potential useful tool for guiding treatment intensities within the early-stage HPV+ OPSCC patients. By applying the threshold defined from the training set, the learned radiomic risk scores could further separate the stage I and stage II HPV+ patients into high- vs. low-risk groups based on disease-free survival. As such, the radiomic biomarker presented in this study could potentially help to distinguish patients within the current AJCC eighth edition definition of low risk as to which patients will benefit from treatment deintensification vs. those who will not. The findings of this study are consistent with a recently published study, where a histology-based imaging biomarker (MuNi) was found to be associated with survival for stage I and stage II patients (34). Noticeably, the prognostic radiomic features identified in this study are mainly from peritumoral compartments. One possible explanation for this is that the peritumoral radiomic features are associated with tumor-infiltrating HPV-specific immune responses prior to treatment, which have been more commonly found floating around the tumor without actual penetration or action into the tumor core and are strongly associated with prognosis (35, 36).

In the third experiment, we developed a radiomic nomogram M_p+RRS_ for HPV+ DFS prediction. Currently, the conventional TNM and AJCC staging systems are routinely used for risk stratification and prognosis estimation. They reflect tumor size (T), lymph node status (N), and cancer metastasis (M). However, these staging factors could not capture the intratumor heterogeneity, which has been shown to be a significant prognostic factor. The radiomic nomogram combined the pathologic staging information with the radiomic features extracted from the entire tumor on CT scans, enabling for robust pretreatment survival estimation. Combining the RRS_HPV+_ from experiment 2 with the pathologic T stage resulted in a nomogram that leads to a more individualized prognosis prediction. With refinement and improvement, this type of radiomic approach might guide more tailored treatment for patients with better survival outcome. Compared with risk stratification using only the conventional staging factors, the radiomic nomogram had an improved DFS estimation. A previous study by Fakhry et al. showed that a nomogram integrating clinicopathologic factors (i.e., HPV status, T and N stages) could reliably predict progression-free survival (37), which is in alignment with our results. Based on our results, T3 stage is significantly associated with worse DFS for HPV+ patients, while N1 and N2 stages are significantly associated with worse DFS for HPV− patients in both univariable and multivariable analyses. With regard to the radiomic nomogram for head and neck cancer, Zhang et al. built multiparametric MRI-based radiomic nomograms for predicting nasopharyngeal carcinoma prognosis and obtained C-index of 0.776 for PFS prediction (38). Yuan et al. proved that a nomogram consisting of MRI radiomic signatures and TNM stage could better predict head and neck cancer prognosis with a C-index of 0.72 on the validation set (39). The prognostic performance difference between our new CT-based nomogram and the MRI-based nomogram may be on the higher image resolution offered by multiparametric MRI, although CT tends to be used more routinely compared with MRI for head and neck cancer. We also noted that both patient cohorts from Zhang et al. (38) and Yuan et al. (39) comprised a majority of advanced stage head and neck cancer patients (100% and 70.6%), while our training and validation cohorts consist of only 17% and 26.6% of stage III and no stage IV OPSCC patients. This may also influence the performance of the model since intratumor heterogeneity could be more easily captured within those aggressive tumors.

Our study does have several limitations. First, the prognostic biomarker validation on a single cohort was done in a retrospective manner. Second, we predicted neither benefit of existing treatments for the two populations nor treatment response within the individual AJCC eighth edition-defined stage groups. These aims will be part of our future study involving large multisite and multimodality evaluation of radiomic signatures in predicting treatment response for the two populations. Third, we acknowledge the limitation of our dataset from the TCIA in terms of HPV status based on p16 testing which may not accurately reflect the true transcriptionally active HPV status, at least for a small percentage of patients. Nonetheless, this study demonstrates that CT radiomic features could, in theory, complement the existing p16 testing method in distinguishing HPV status.

Despite the aforementioned limitations, this study is the first to show the role of combined intratumoral and peritumoral radiomic features in predicting HPV status of OPSCC patients. It is also the first study to incorporate both radiomic signatures and corresponding nomograms for prognosis prediction for HPV+ and HPV− patients. If confirmed in prospective clinical trials, this radiomic nomogram pipeline could enrich the existing AJCC eighth staging systems for risk-stratifying OPSCC patients. One can imagine a strategy where numerous sources of data go into predictive models for patient care. Especially attractive here is that all patients received pretreatment cross-sectional CT scans so the data are already garnered in digitized form, easily available for radiomics-based nomograms for prognosis prediction.

## Data Availability Statement

The raw data supporting the conclusions of this article will be made available by the authors, without undue reservation.

## Ethics Statement

The studies involving human participants were reviewed and approved by Cleveland Clinic. The patients/participants provided their written informed consent to participate in this study.

## Author Contributions

BS: conceptualization, data curation, formal analysis, methodology, investigation, writing—original draft, and writing—review and editing. KY: conceptualization, investigation, and writing—review and editing. JG: writing—review and editing. CL: writing—review and editing. LL: formal analysis and investigation. NB: writing—review and editing. CK: writing—review and editing. PT: resources and investigation. PF: conceptualization, investigation, and writing—review and editing. SK: resources, investigation, and writing—review and editing. JSL: resources, investigation, and writing—review and editing. AM: conceptualization, methodology, investigation, supervision, funding acquisition, project administration, and writing—review and editing. All authors contributed to the article and approved the submitted version.

## Funding

Research**** reported in this publication was supported by the National Cancer Institute under award numbers 1U24CA199374-01, R01 CA24999201A1, R01CA202752-01A1, R01CA208236-01A1, R01CA216579-01A1, R01CA220581-01A1, 1U01CA239055-01, 1U01CA248226-01, and 1U54CA254566-01; National Heart, Lung and Blood Institute 1R01HL15127701A1; National Institute for Biomedical Imaging and Bioengineering 1R43EB028736-01; National Center for Research Resources under award number 1 C06 RR12463-01; VA Merit Review Award IBX004121A from the United States Department of Veterans Affairs Biomedical Laboratory Research and Development Service; the Office of the Assistant Secretary of Defense for Health Affairs, through the Breast Cancer Research Program (W81XWH-19-1-0668), the Prostate Cancer Research Program (W81XWH-15-1-0558, W81XWH-20-1-0851), the Lung Cancer Research Program (W81XWH-18-1-0440, W81XWH-20-1-0595), and the Peer Reviewed Cancer Research Program (W81XWH-18-1-0404); the Kidney Precision Medicine Project (KPMP) Glue Grant; the Ohio Third Frontier Technology Validation Fund; the Clinical and Translational Science Collaborative of Cleveland (UL1TR0002548) from the National Center for Advancing Translational Sciences (NCATS) component of the National Institutes of Health and NIH roadmap for Medical Research; and the Wallace H. Coulter Foundation Program in the Department of Biomedical Engineering at Case Western Reserve University. KY was supported by the Computational Genomic Epidemiology of Cancer (CoGEC) Program at Case Comprehensive Cancer Center (T32CA094186).

## Author Disclaimer

The content is solely the responsibility of the authors and does not necessarily represent the official views of the National Institutes of Health, the U.S. Department of Veterans Affairs, the Department of Defense, or the United States Government.

## Conflict of Interest

AM is an equity holder in Elucid Bioimaging and in Inspirata Inc. In addition, he has served as a scientific advisory board member for Inspirata Inc., AstraZeneca, Bristol Myers Squibb, and Merck. Currently, he serves on the advisory board of Aiforia Inc. He also has sponsored research agreements with Philips, AstraZeneca, Boehringer Ingelheim, and Bristol Myers Squibb. His technology has been licensed to Elucid Bioimaging. He is also involved in a NIH U24 grant with PathCore Inc. and three different R01 grants with Inspirata Inc. SK is a consultant for Merck and Regeneron, receives research support from Merck and Bristol Myers Squibb, and reports honoraria from UpToDate.

The remaining authors declare that the research was conducted in the absence of any commercial or financial relationships that could be construed as a potential conflict of interest.

## Publisher’s Note

All claims expressed in this article are solely those of the authors and do not necessarily represent those of their affiliated organizations, or those of the publisher, the editors and the reviewers. Any product that may be evaluated in this article, or claim that may be made by its manufacturer, is not guaranteed or endorsed by the publisher.

## References

[1] Stein AP Saha S Kraninger JL Swick AD Yu M Lambert PF. Prevalence of Human Papillomavirus in Oropharyngeal Cancer: A Systematic Review. Cancer J (2015) 21:138–46. 10.1097/PPO.0000000000000115 PMC4459520 26049691

[2] Ang KK Harris J Wheeler R Weber R Rosenthal DI Nguyen-Tân PF. Human Papillomavirus and Survival of Patients With Oropharyngeal Cancer. N Engl J Med (2010) 363:24–35. 10.1056/NEJMoa0912217 20530316 PMC2943767

[3] Fakhry C Westra WH Li S Cmelak A Ridge JA Pinto H. Improved Survival of Patients With Human Papillomavirus-Positive Head and Neck Squamous Cell Carcinoma in a Prospective Clinical Trial. J Natl Cancer Inst (2008) 100:261–9. 10.1093/jnci/djn011 18270337

[4] Machczyński P Majchrzak E Niewinski P Marchlewska J Golusiński W. A Review of the 8th Edition of the AJCC Staging System for Oropharyngeal Cancer According to HPV Status. Eur Arch Otorhinolaryngol (2020) 277:2407–12. 10.1007/s00405-020-05979-9 PMC7410862 32342197

[5] Howard J Dwivedi RC Masterson L Kothari P Quon H Holsinger FC. De-Intensified Adjuvant (Chemo)Radiotherapy Versus Standard Adjuvant Chemoradiotherapy Post Transoral Minimally Invasive Surgery for Resectable HPV-Positive Oropharyngeal Carcinoma. Cochrane Database Syst Rev (2018) 12:CD012939. 10.1002/14651858.CD012939.pub2 30550641 PMC6517188

[6] Amin MB Greene FL Edge SB Compton CC Gershenwald JE Brookland RK. The Eighth Edition AJCC Cancer Staging Manual: Continuing to Build a Bridge From a Population-Based to a More “Personalized” Approach to Cancer Staging. CA Cancer J Clin (2017) 67:93–9. 10.3322/caac.21388 28094848

[7] Yom SS Torres-Saavedra P Caudell JJ Waldron JN Gillison ML Xia P. Reduced-Dose Radiation Therapy for HPV-Associated Oropharyngeal Carcinoma (NRG Oncology HN002). J Clin Oncol (2021) 39:956–65. 10.1200/JCO.20.03128 PMC8078254 33507809

[8] Würdemann N Wagner S Sharma SJ Prigge E-S Reuschenbach M Gattenlöhner S. Prognostic Impact of AJCC/UICC 8th Edition New Staging Rules in Oropharyngeal Squamous Cell Carcinoma. Front Oncol (2017) 7:129. 10.3389/fonc.2017.00129 28713770 PMC5491554

[9] Becker C Hofauer BG Mansour N Ketterer MC Schulz T Knopf A. The 8th Edition of the TNM Staging System-A Curse or a Bfor Oropharyngeal Carcinoma? HNO (2021) 69(2):89–94. 10.1007/s00106-020-00875-4 32385531

[10] Kwan JYY Su J Huang SH Ghoraie LS Xu W Chan B. Radiomic Biomarkers to Refine Risk Models for Distant Metastasis in HPV-related Oropharyngeal Carcinoma. Int J Radiat Oncol Biol Phys (2018) 102(4):1107–16. 10.1016/j.ijrobp.2018.01.057 29506884

[11] Wong AJ Kanwar A Mohamed AS Fuller CD. Radiomics in Head and Neck Cancer: From Exploration to Application. Transl Cancer Res (2016) 5:371–82. 10.21037/tcr.2016.07.18 PMC6322843 30627523

[12] Beig N Khorrami M Alilou M Prasanna P Braman N Orooji M. Perinodular and Intranodular Radiomic Features on Lung CT Images Distinguish Adenocarcinomas From Granulomas. Radiology (2019) 290:783–92. 10.1148/radiol.2018180910 PMC6394783 30561278

[13] Braman N Prasanna P Whitney J Singh S Beig N Etesami M. Association of Peritumoral Radiomics With Tumor Biology and Pathologic Response to Preoperative Targeted Therapy for HER2 (ERBB2)-Positive Breast Cancer. JAMA Netw Open (2019) 2(4):e192561. 10.1001/jamanetworkopen.2019.2561 31002322 PMC6481453

[14] Clark K Vendt B Smith K Freymann J Kirby J Koppel P. The Cancer Imaging Archive (TCIA): Maintaining and Operating a Public Information Repository. J Digit Imaging (2013) 26:1045–57. 10.1007s10278-013-9622-7 10.1007/s10278-013-9622-7 PMC3824915 23884657

[15] Mohiuddin K Haneuse S Sofer T Gill R Jaklitsch MT Colson YL. Relationship Between Margin Distance and Local Recurrence Among Patients Undergoing Wedge Resection for Small (≤2 cm) Non-Small Cell Lung Cancer. J Thorac Cardiovasc Surg (2014) 147:1169–75; discussion 1175-1177. 10.1016/j.jtcvs.2013.11.056 24507406

[16] Haralick RM Shanmugam K Dinstein I. Textural Features for Image Classification In: IEEE Transactions on Systems, Man, and Cybernetics, vol. SMC-3, no. 6. (1973) pp. 610–21 10.1109/TSMC.1973.4309314

[17] Gillett WD. Image Classification Using Laws’ Texture Energy Measures. All Computer Science and Engineering Research. (1987). Report Number: WUCS-87-25.

[18] Fogel I Sagi D. Gabor Filters as Texture Discriminator. Biol Cybern (1989) 61:103–13. 10.1007/BF00204594

[19] Prasanna P Tiwari P Madabhushi A. Co-Occurrence of Local Anisotropic Gradient Orientations (CoLlAGe): A New Radiomics Descriptor. Sci Rep (2016) 6:37241. 10.1038/srep37241 27872484 PMC5118705

[20] Shirzad MB Keyvanpour MR. A Feature Selection Method Based on Minimum Redundancy Maximum Relevance for Learning to Rank. AI Robotics (IRANOPEN) (2015) 2015:1–5. 10.1109/RIOS.2015.7270735

[21] Beig N Bera K Prasanna P Antunes J Correa R Singh S. Radiogenomic-Based Survival Risk Stratification of Tumor Habitat on Gd-T1w MRI Is Associated With Biological Processes in Glioblastoma. Clin Cancer Res (2020) 26:1866–76. 10.1158/1078-0432.CCR-19-2556 PMC7165059 32079590

[22] Vickers AJ van Calster B Steyerberg EW. A Simple, Step-by-Step Guide to Interpreting Decision Curve Analysis. Diagn Progn Res (2019) 3:18. 10.1186/s41512-019-0064-7 31592444 PMC6777022

[23] Taberna M Mena M Pavón MA Alemany L Gillison ML Mesía R. Human Papillomavirus-Related Oropharyngeal Cancer. Ann Oncol (2017) 28:2386–98. 10.1093/annonc/mdx304 28633362

[24] Singhi AD Westra WH. Comparison of Human Papillomavirus *in Situ* Hybridization and P16 Immunohistochemistry in the Detection of Human Papillomavirus-Associated Head and Neck Cancer Based on a Prospective Clinical Experience. Cancer (2010) 116:2166–73. 10.1002/cncr.25033 20186832

[25] Leijenaar RT Bogowicz M Jochems A Hoebers FJ Wesseling FW Huang SH. Development and Validation of a Radiomic Signature to Predict HPV (P16) Status From Standard CT Imaging: A Multicenter Study. Br J Radiol (2018) 91:20170498. 10.1259/bjr.20170498 29451412 PMC6223271

[26] Bagher-Ebadian H Lu M Siddiqui F Ghanem AI Wen N Wu Q. Application of Radiomics for the Prediction of HPV Status for Patients With Head and Neck Cancers. Med Phys (2020) 47:563–75. 10.1002/mp.13977 31853980

[27] Chernock RD. Morphologic Features of Conventional Squamous Cell Carcinoma of the Oropharynx: “Keratinizing” and “Nonkeratinizing” Histologic Types as the Basis for a Consistent Classification System. Head Neck Pathol (2012) 6 Suppl 1:S41–47. 10.1007/s12105-012-0373-4 PMC3394167 22782222

[28] Westra WH. The Morphologic Profile of HPV-Related Head and Neck Squamous Carcinoma: Implications for Diagnosis, Prognosis, and Clinical Management. Head Neck Pathol (2012) 6 Suppl 1:S48–54. 10.1007/s12105-012-0371-6 PMC3394160 22782223

[29] Chernock RD El-Mofty SK Thorstad WL Parvin CA Lewis JS. HPV-Related Nonkeratinizing Squamous Cell Carcinoma of the Oropharynx: Utility of Microscopic Features in Predicting Patient Outcome. Head Neck Pathol (2009) 3:186–94. 10.1007/s12105-009-0126-1 PMC2811624 20596971

[30] Leijenaar RTH Carvalho S Hoebers FJP Aerts HJWL van Elmpt WJC Huang SH. External Validation of a Prognostic CT-Based Radiomic Signature in Oropharyngeal Squamous Cell Carcinoma. Acta Oncol (2015) 54:1423–9. 10.3109/0284186X.2015.1061214 26264429

[31] Vallières M Kay-Rivest E Perrin LJ Liem X Furstoss C Aerts HJWL. Radiomics Strategies for Risk Assessment of Tumour Failure in Head-and-Neck Cancer. Sci Rep (2017) 7:10117. 10.1038/s41598-017-10371-5 28860628 PMC5579274

[32] Aerts HJWL Velazquez ER Leijenaar RTH Parmar C Grossmann P Carvalho S. Decoding Tumour Phenotype by Noninvasive Imaging Using a Quantitative Radiomics Approach. Nat Commun (2014) 5:4006. 10.1038/ncomms5006 24892406 PMC4059926

[33] Oguejiofor K Hall J Slater C Betts G Hall G Slevin N. Stromal Infiltration of CD8 T Cells is Associated With Improved Clinical Outcome in HPV-Positive Oropharyngeal Squamous Carcinoma. Br J Cancer (2015) 113:886–93. 10.1038/bjc.2015.277 PMC4578081 26313665

[34] Koyuncu CF Lu C Bera K Zhang Z Xu J Toro P. Computerized Tumor Multinucleation Index (MuNI) is Prognostic in P16+ Oropharyngeal Carcinoma. J Clin Invest (2021) 131(8):e145488. 10.1172/JCI145488 PMC8075166 33651718

[35] Sinha P Lewis JS Kallogjeri D Nussenbaum B Haughey BH. Soft Tissue Metastasis in P16-Positive Oropharynx Carcinoma: Prevalence and Association With Distant Metastasis. Oral Oncol (2015) 51:778–86. 10.1016/j.oraloncology.2015.05.004 26033471

[36] Hanoteau A Newton JM Krupar R Huang C Liu H-C Gaspero A. Tumor Microenvironment Modulation Enhances Immunologic Benefit of Chemoradiotherapy. J Immunother Cancer (2019) 7:10. 10.1186/s40425-018-0485-9 30646957 PMC6332704

[37] Fakhry C Zhang Q Nguyen-Tân PF Rosenthal DI Weber RS Lambert L. Development and Validation of Nomograms Predictive of Overall and Progression-Free Survival in Patients With Oropharyngeal Cancer. J Clin Oncol (2017) 35:4057–65. 10.1200/JCO.2016.72.0748 PMC5736236 28777690

[38] Zhang B Tian J Dong D Gu D Dong Y Zhang L. Radiomics Features of Multiparametric MRI as Novel Prognostic Factors in Advanced Nasopharyngeal Carcinoma. Clin Cancer Res (2017) 23:4259–69. 10.1158/1078-0432.CCR-16-2910 28280088

[39] Yuan Y Ren J Shi Y Tao X. MRI-Based Radiomic Signature as Predictive Marker for Patients With Head and Neck Squamous Cell Carcinoma. Eur J Radiol (2019) 117:193–8. 10.1016/j.ejrad.2019.06.019 31307647

