# Supplementary material for: Radiomic Features Associated With HPV Status on Pretreatment Computed Tomography in Oropharyngeal Squamous Cell Carcinoma Inform Clinical Prognosis
Source: Front Oncol. 2021 Sep 7;11:744250. doi: 10.3389/fonc.2021.744250 (PMC8454409; doi:10.3389/fonc.2021.744250)
Supplement: Supplementary file 1 [file DataSheet_1.docx]

**Table S1:** Summary of the radiomic features extracted

| Region of Interest (ROI) | Feature category | Window size in pixels (Intratumoral) or annular ring (Peritumoral) | Number of statistics | Number of features extracted (total) | Total number of features |
| --- | --- | --- | --- | --- | --- |
| Region within the tumor boundary (Intratumoral) | Gray level intensity (4) | 3,5,7,9 | 4 | 4×4×4 (64) | 664 |
|  | Intensity gradient (10) | 3 | 4 | 10×1×4 (40) |  |
|  | Gabor frequency, orientations (7) | 3,5,7,9 | 4 | 7×4×4 (112) |  |
|  | Laws’ texture energy (5) | 3,5 | 4 | 3^2^×4+5^2^×4 (136) |  |
|  | Haralick textures (13) | 3,5,7,9 | 4 | 13×4×4 (208) |  |
|  | Co-occurrence of Gradient Orientations (CoLlAGe) (13) | 3,5 | 4 | 13×2×4 (104) |  |
| Annular rings outside the tumor boundary (Peritumoral) | Haralick textures (13) | 0-5mm, 5-10mm, 10-15mm | 5 | 13×3×5 (195) | 1485 |
|  | CoLlAGe (13) | 0-5mm, 5-10mm, 10-15mm | 5 | 13×3×5 (195) |  |
|  | Gabor frequency, orientations (48) | 0-5mm, 5-10mm, 10-15mm | 5 | 48×3×5 (720) |  |
|  | Laws’ texture energy (25) | 0-5mm, 5-10mm, 10-15mm | 5 | 25×3×5 (375) |  |


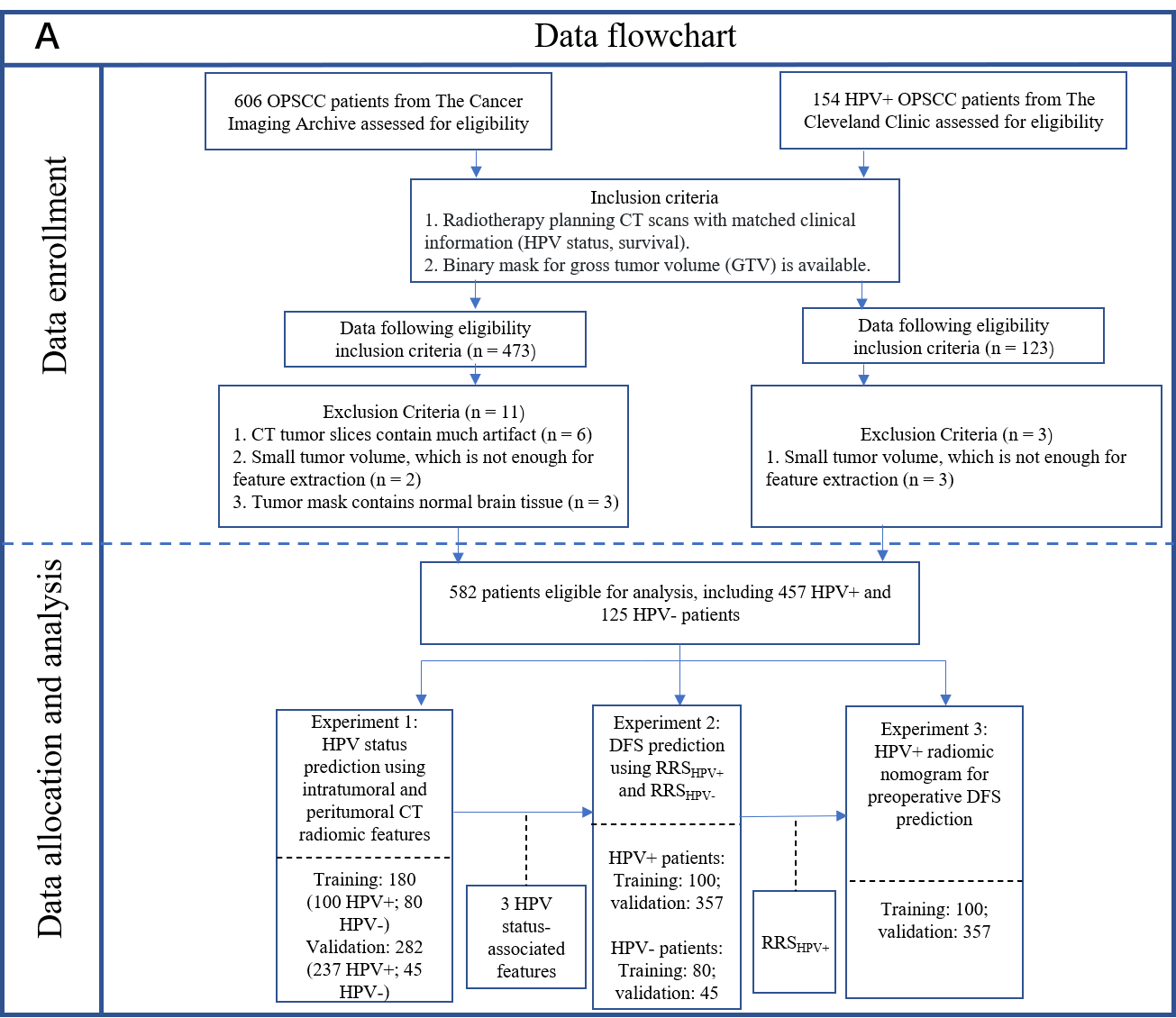


**Figure S1**: Diagram of the overall data enrollment, allocation and analysis flowchart.


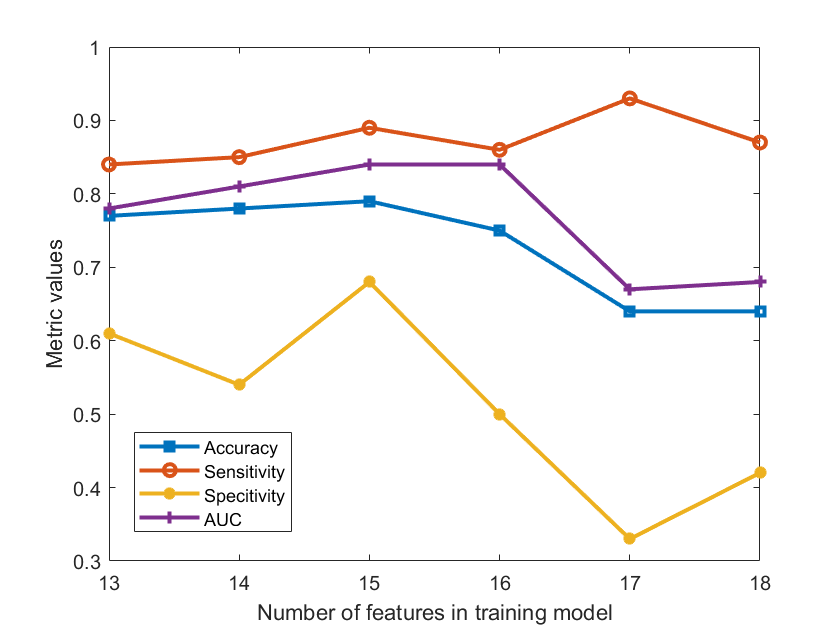


**Figure S2**: Classification metrics changing by different numbers of features selected in training set. The accuracy, specificity and AUC were the highest when top 15 features were selected for the model.


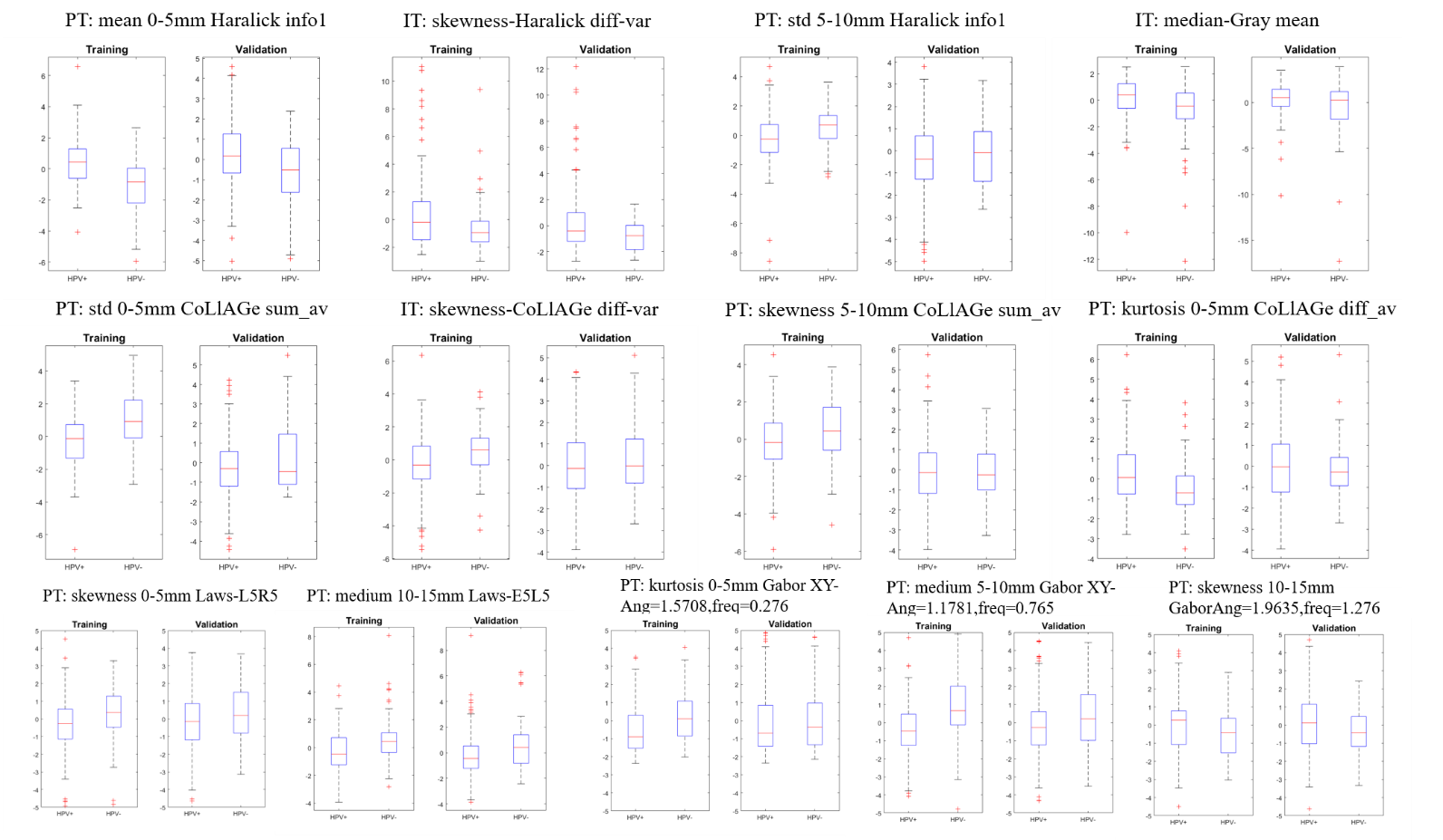


**Figure S3**: Box plots for the remaining selected features other than the best intratumoral and peritumoral feature provided in the main text.


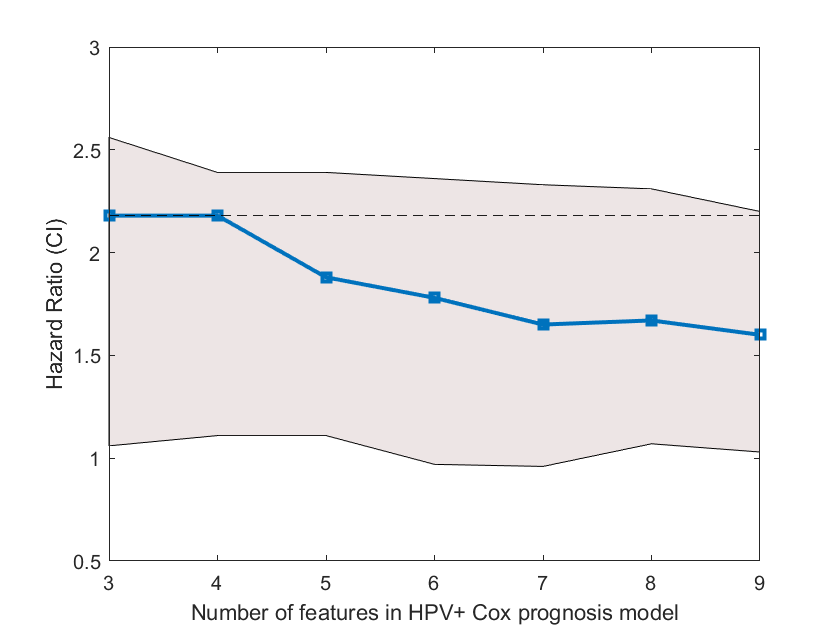


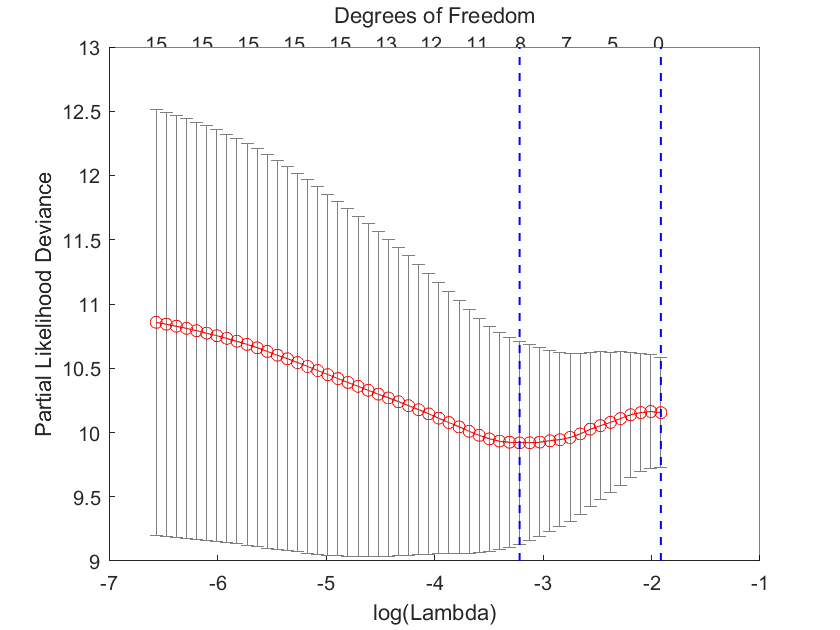


**Figure S4**: Hazard ratio with 95% confidence interval changing by different number of features selected in the Cox model for HPV+ patients (top). Elastic net regularization process with partial likelihood deviance plot (bottom).


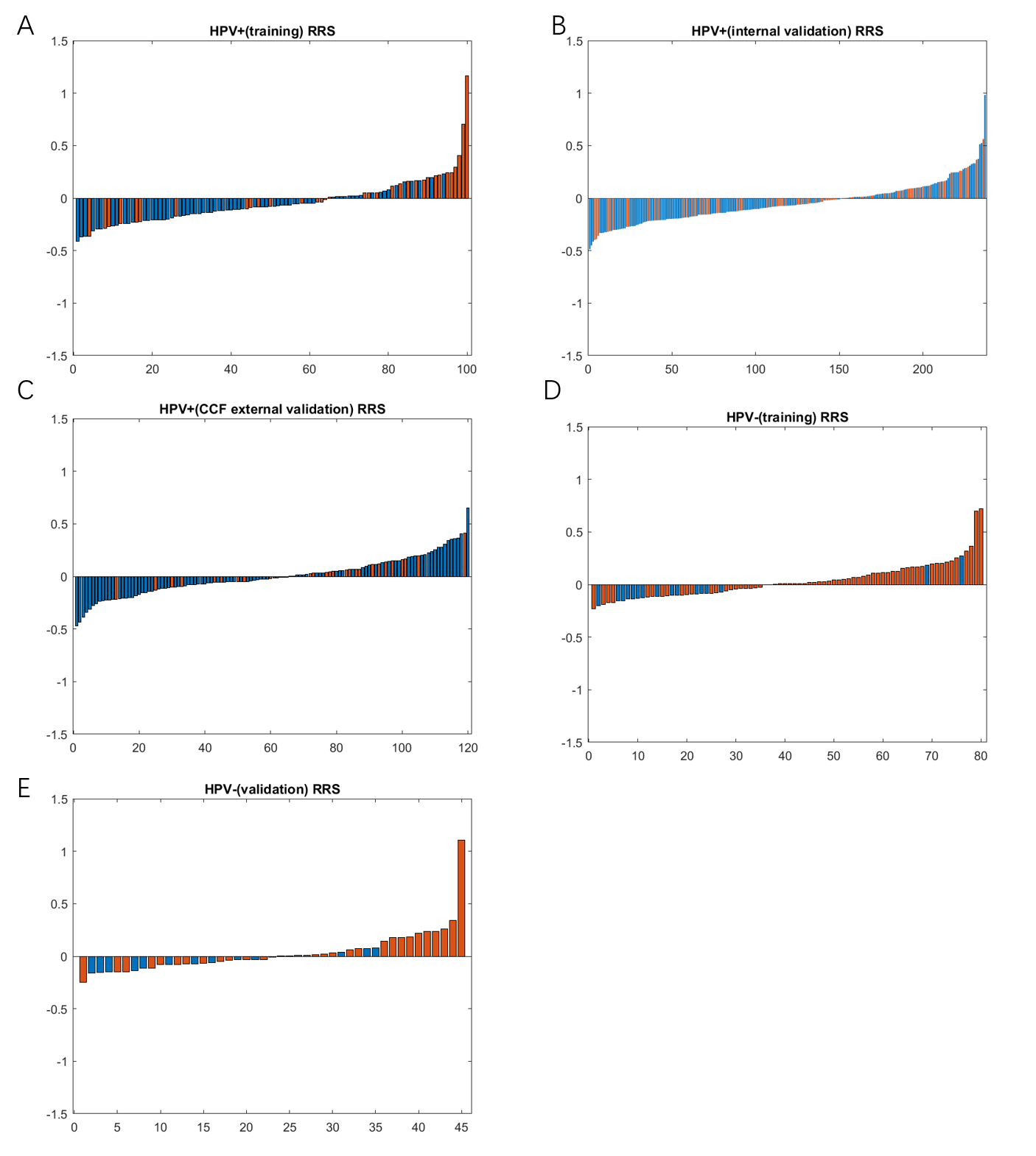


**Figure S5**. RRS_HPV+_ for each patient in TCIA training (A), TCIA internal validation set (B) and CCF external validation set (C). RRS_HPV-_ for each patient in training (D) and validation set (E). Blue bars indicate censored patients (survived without disease recurrence) and orange bars indicate those patients who had the recurrence or died. Recurrence rate for HPV- patients is higher than that of HPV+ patients. RRS_HPV-_was able to classify most of the patients who had the recurrence or died into high-risk.


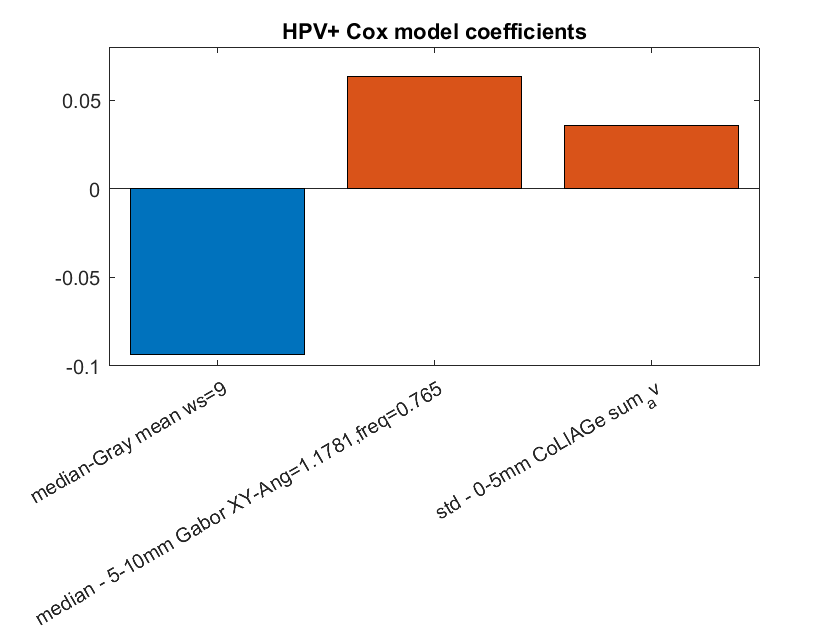


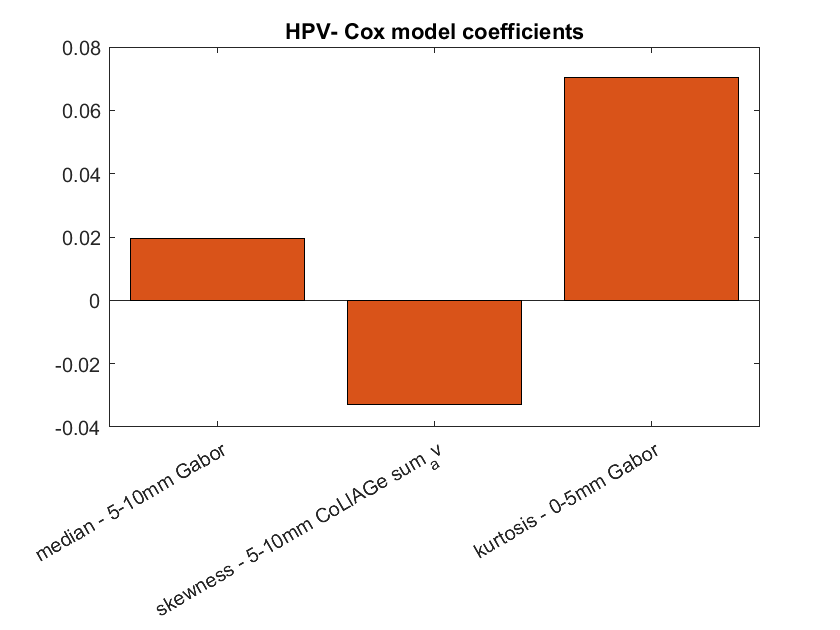


**Figure S6**. Cox model coefficients for HPV+ (top) and HPV- (bottom) patients. Orange bars represent coefficients for peritumoral features while blue represent intratumoral features.

**Appendix E1**

**Section 1: Peritumoral masks generation**

For all patients, peritumoral masks were dilated a distance of 15 mm from the corresponding intratumoral masks in a 2-dimensional fashion. For each CT axial slice with tumor, the number of pixels dilated in each peritumoral mask were calculated as follows:

$$\# pixels dilated=\frac{15}{pixel size(in mm)}$$

During this peritumoral feature extraction process, additional consideration was taken to get rid of the region with air (<-900 HU). To avoid any edge artifacts that might arise during feature extraction, the filtered ‘dead’ pixels of the CT scan were substituted by using an averaging filter across its 9 × 9 neighborhood.

**Section 2: Expanded description of the extracted radiomic features**

1. *Gray level intensity features*: Statistics were calculated on the gray level intensity values within a neighborhood of 3 by 3,5 by 5,7 by 7 and 9 by 9.
2. *Intensity gradient-based features*: Statistics were calculated on the image gradients:

$$\nabla f= \left[ \begin{matrix} \frac{\partial f}{\partial x} \\ \frac{\partial f}{\partial y} \end{matrix} \right]$$

where:

$\frac{\partial f}{\partial x}$ is the derivative with respect to x (gradient in the x direction) and $\frac{\partial f}{\partial y}$ is the derivative with respect to y (gradient in the y direction).

1. *Laws*: 2-dimensional Laws filters are derived by computing the outer product of combinations of the following 1-dimensional filter vectors focused on different texture patterns:

Level (L5) – detects smoothness of intensity values. L5 = [1 4 6 4 1]

Edge (E5) – detects edges between regions with abrupt changes in intensity. E5 = [-1 -2 0 2 1]

Spot (S5) – detects speckled enhancement patterns. S5 = [-1 0 2 0 -1]

Wave (W5) – detects regularly oscillating local intensity patterns. W5 = [-1 2 0 -2 1]

Ripple (R5) – detects oscillating intensity patterns centered at region of extreme intensity. R5 = [ 1 -4 6 -4 1]

To obtain a feature vector, convolution is performed on the filters and the images within a window size neighborhood followed by summing up all the values. Features are named by the combination of filters applied in the y and x axes, e.g. L5E5 is the product of a level detection filter in the y axis and an edge detection filter in the x axis.

1. *Gabor*: 2-dimensional Gabor filters are computed by modulating a Gaussian kernel function with one of 48 sinusoidal plane waves. Each sinusoidal plane wave corresponds to a unique combination of one of four spatial wavelengths (2 pixels, 4 pixels, 8 pixels, 12 pixels) and one of seven orientations (22.5°, 45°, 67.5°, 90°, 112.5°, 135°, 157.5°). Each Gabor filter is then convolved with the original image and values corresponding to filter response within the region of interest are concatenated.
2. *Haralick features*: The following Haralick gray level co-occurrence matrix (GLCM) descriptors were computed: entropy, energy, inertia, inverse difference moment, correlation, information measure of correlation 1, information measure of correlation 2, sum average, sum variance, sum entropy, difference average, difference variance, and difference entropy. GLCM statistics were concatenated within a window size neighborhood, yielding 13 descriptor vectors per region.
3. *Co-occurrence of local anisotropy gradients (CoLlAGe)*: An image’s intensity gradients in the x and y direction are computed. Within a window size neighborhood, the dominant intensity gradient orientation (between 0°-360°) is computed via principal component analysis, resulting in a 2D array of equal size with the dominant gradient orientation value centered at the corresponding pixel of the original image. Metrics of the co-occurrence matrix are then applied to this gradient orientation image in the same manner as described above for Haralick GLCM features. The resulting 13 CoLlAGe descriptors quantify the homogeneity of intensity gradient directionality within an image.

**Section 3: CT imaging parameters** The CT images for the TCIA cohort were acquired from one of the following CT scanners: General Electric Discovery ST; General Electric Lightspeed Plus; Toshiba Medical Systems Aquillion ONE. CT scans were acquired in helical mode with a slice thickness of 2.5 mm (General Electric) or 2 mm (Toshiba), at 120 kVp and 300 mAs tube current. Image resolution was 1 mm for all the scans.

The CT images for the CCF cohort were acquired from either The General Electric Medical System or The Siemens Medical System. CT scans were acquired in helical mode with a slice thickness of 3 mm, at 120 kVp and 235 mAs tube current. Image resolution is between 0.4 mm - 0.5 mm for most of the patients, with image matrix of 512 × 512.
